# Thirty-First Annual Meeting of the American Dental Association

**Published:** 1891-09

**Authors:** N. S. Hoff

**Affiliations:** Ann Arbor, Michigan


### Society Proceedings.

### The Thirty-First Annual Meeting of the American Dental Association.

SARATOGA SPRINGS, N. Y., AUGUST 4, 1891.

Reported by N. S. Hoff, D.D.S., Ann Arbor, Michigan.

The meeting was called to order at 10:15 A. M. in the City Hall council room, by President A. W. Harlan. All the officers except E. T. Darby and S. G. Perry, of the executive committee, were present and in their places.

The usual preliminary reading of minutes, roll call, etc., were duly disposed of, also the secretary, treasurer and executive committee reports.

At the session last year a committee was appointed to confer with the authorities at Washington and urge the appointment of dental surgeons to the army and navy. This committee reported a conference with the surgeon general and read a communication from him to the effect, that owing to the peaceful times and almost complete abolishment of frontier life, there was no need of an army dental surgeon as the troops were located in towns and cities where such services could be secured readily, and that the small numbers of the troops that were out of the reach of such services were continually on the march, so it would be impracticable to appoint a dental surgeon for them. Further, the expense to the army, and opportunity for extravagance and abuse of the privilege, would more than counterbalance any desirable end that might be sought by such appointments. The committee therefore desired to submit an unfavorable report, which was accepted.

The committee on dental legislation made a report and urged as a solution of the differences between college faculties and State examining boards, that a committee should be appointed from each State board where a college was located, to assist at the examination of candidates for graduation in connection with the college faculty, and such examination and indorsement was to be accepted as final in any State. The report was accepted, but no further recommendation or action taken, although many could not endorse the wisdom of such a proceeding.

The committee appointed to assist the New Hampshire dentists in defending their State law, reported that the supreme court had decided that the law was unconstitutional in that it made personal distinctions merely on the ground of residence. The committee reported that they had nothing to do as the dentists of New Hampshire had wisely decided to drop all further contest and concentrate their efforts and secure a new and better law.

President A. W. Harlan then read his annual address in which he spoke feelingly of the death of Dr. Wm. H. Atkinson, the first president of the association; Dr. Edward Maynard, the well-known inventor dentist, and the one who perfected the non-cohesive gold filling system, and Dr. J. W. White, President of the S. S. White Dental Manufacturing Co., and the successful and respected Editor of the *Dental Cosmos.*

The president advised the abolishment of all standing committees, transferring the miscellaneous business matters of the association to an annually elected business board, or executive committee; the idea being to secure the full and uninterrupted time of the meeting for the reading and discussion of scientific papers.

Advised that the officers of the sections be changed oftener in order that more variety, and possibly improvement, in the quality of work done might be secured.

Advised such change of the constitution as would allow the association, or executive committee, by majority vote, to change the time and place of meeting, with the idea that if it should at any time seem desirable to hold a meeting in the south an agreeable season could be selected.

Advised the appointment of committees in suitable localities to assist in the organization of local societies in new States, cities, towns or districts where none now exist. This committee to be supervisory and report to this association.

Congratulated the association on the accomplishment, with so little apparent disturbance, of the extended college course of study required for graduation, and the influence it had exerted to bring this to pass.

After reading the president's address, on motion, a committee was appointed to which it was referred, with the request that it make such report for the action of the society as seemed expedient. This committee subsequently reported extended amendments to the constitution embracing not only the ideas advanced by the president, but some other seemingly desirable amendments, which under the rules will lie over until next year for action.

EVENING SESSION.

SECTION VI. Physiology and etiology was called and Dr. H. A. Smith reported as chairman. In continuation of the report from last year, the work of the committee had established the fact that implantation was successful for from three to five years. No person, so far as known, has been inoculated with disease through the practice of implantation. No operation for restoration of lost dental organs is so æsthetic or useful, and for these reasons it is to be commended, even though its existence be as brief as three years. The success of implantation in connection with antiseptic agents has revived interest in the subject of transplantation, and a combination of implanting and transplanting will often enable the skillful practitioner to utilize the socket of a tooth lost from pyorrhœa alveolaris or other cause.

In conclusion the committee beg to report that the profession is justified in performing this operation in suitable cases, inasmuch as the chances of success are favorable when a healthy tooth is implanted and the operation skillfully made.

Dr. Patrick, to whom the work of examining the prehistoric crania of the country had been delegated by this section, reported that 2,096 skulls have been examined but the reports came in too late for classification for this meeting. The crania at Harvard were examined by Dr. Andrews; at Philadelphia, by Dr. Peirce; at St. Louis, by Drs. McKellops and Fuller; at Chicago, by Drs. Ottofy and Davis. Other collections were being examined in varions parts of the country. Some valuable collections could not be examined except by the curators of the museums where they are located, and as this would involve expense a request was asked for more money to carry on this work. At a subsequent meeting five hundred dollars ($500) was voted in addition to a similar amount voted last year. Dr. Patrick exhibited the blank forms used with the directions for taking the measurements and requested that any one living near a collection should send to him for the blanks and make the examinations. He also exhibited some of the charts filled in with the data obtained from examinations already held.

Dr. Eugene S. Talbot read a paper entitled, "Mouth Breathing not the Cause of Contracted Jaws and High Vaults."

Mouth breathing was unknown among the earlier races and is not observed among the present uncivilized races. Dr. Delavan (see *International Dental Journal*, January, 1891) thinks mouth breathing compels the constant dropping of the lower jaw, producing by the stretching of the muscles a lateral pressure on the bones of the face which results in crushing or compressing them. Other authors attribute the compression to the tension put upon the buccinator muscle alone. Dr. Talbot describes the anatomical peculiarities of the bones of the face at considerable length in regard to their construction to resist force, etc., at too great length for our abstract. The hard palate does not assume the normal shape until the twelfth year, or after the teeth assume their positions in the arch. And by measurements made on a large number of skulls and models (over 6,000) the height of the vault measured from a line drawn across it between the second bicuspid and first molars at the margin of the alveolar process varies from one-fourth of an inch (the lowest) to one inch, the highest observed. The alveolar process is developed for the support of the teeth and it conforms to the positions taken by the teeth, while the maxilla is not appreciably disturbed by any abnormal positions taken by the teeth. Many cases of contracted arches were observed unaccompanied by mouth breathing; on the contrary in cases of mouth breathers he observed many normal arches. Mouth breathing sometimes begins early in life, but contracted arches seldom occur until the seventh to the tenth year. These contracted arches always assume the V-shape, saddle shape, or a modification of one or the other, or the two.

There are more contracted jaws among the low than the high vaults, but the deformity in the high vault is more marked. In the V-shaped arch the contraction begins at the first molar and extends to the incisors, projecting the process. While in the saddle-shaped the bicuspids alone are crowded inward and there is no projection of the process or incisors. The contracted hard palate is also associated with the V-shaped and never with the saddle-shaped deformity. The high vault is never seen in the deciduous set of teeth.

The buccinator muscle is used for blowing and keeping food under the teeth when eating, and during sleep there can be no tension on this muscle. As this muscle in its attachment only extends as far forward as the first bicuspid it could not possibly be a factor in producing the V-shaped arch ; neither could the orbicularis oris, as the action of this muscle would be to drive the incisors inward and backward, directly antagonizing any possible tendency of the buccinator to do this. The roots of the teeth have nothing to do with determining the position of the teeth, or the shape of the arch, as they are developed largely after the tooth has taken its position in the arch. If the muscles were active in producing the deformity it would be uniform on both sides of the mouth, but such is not the case generally. The muscular action is not sufficient to bend the hard palate by any pressure made upon the teeth. But there is no bending of the bones; the process is one of absorption and deposition, and besides the muscular power of the tongue is sufficient to counteract the muscular power of the facial muscles. Partial irregularities preclude the muscular cause. In most cases the cause of the deformity is the arrest of development of the maxillary bones, due to hereditary influences or disease. An examination of 4,614 cases gives an average height of the arch of 0.58 of an inch. In the saddle-shaped arch the average height was 0.60 of an inch. In the V-shaped arch the average height was 0.55 ot of an inch. In the semi-V-shaped arch the average height was

0.56 of an inch. The height of the vault seems to depend upon the shape of the cranium, or have a uniform relation to it. The impression that the vault of contracted arches is higher than the normal is erroneous, and due to the contracted arch.

The author of the paper exhibited models of twenty-four mouth breathers, from one to twenty-four years of age, none of which showed any special tendency toward contracted arches or high vaults.

DISCUSSION.

Dr. Friederichs does not think an examination of even six thousand cases sufficient to establish a typical arch.

Dr. Frank Abbott would congratulate Dr. Talbot and thought he had satisfactorily established the fact that mouth breathing does not produce the V-shaped or saddle-shaped arches.

Dr. Fillebrown agreed with the essayist in the statement that the buccinator muscles could not be important factors in producing this deformity since there was considerable space between the teeth and the muscles when the lower jaw was dropped to its utmost.

Dr. Horton was surprised to learn that mouth breathing was confined to the civilized races, and he would like more information on this subject.

Dr. Watkins would like to know if mouth breathing after the eruption of the teeth would cause the vaulted arch. Dr. Talbot said he had never seen the V-shaped arch in the jaws of children.

Dr. Peirce: Mouth breathing has not been generally given as the cause of contracted arches. The reason why there is no V-shaped arch in children is that the child's jaw is developed largely before it is born, and when there can be no mechanical pressure exerted to malform it. If the malformation is produced between the ages of eight and twelve years, that is the time when the permanent bicuspids and cuspids are taking their places in the arch, and the contracted arches could easily be produced by the muscular pressure brought to bear upon them by the labial and facial muscles.

Dr. Morgan: It is uncommon to find among the negroes high vaults or contracted arches or irregularities of the teeth, an indication that the malady may predominate among highly civilized people.

Dr. Truman would like to know why the permanent bicuspids are thrown in by the activity of the muscles, and the deciduous molars are not.

Dr. Peirce thought that was due to the fact that the crowns of the temporary teeth had developed so far and taken their normal positions in the arch that the action of the muscles in the babe were not sufficient to produce the contraction, especially as the alveolar process begins to develop as soon as the crown is formed and this would resist the action of the muscles.

Dr. Patrick: The measurements given in this paper are worth nothing so far as they have been used to establish a fact. Averages are not scientific, they are tentative, speculative. Every additional measurement will change and destroy the average. Averages are useless. These measurements were not made of any single race or class of people or they might be useful in establishing scientific facts. Averages destroy individuality and prevent classification. Measurements are good, but averages prove nothing. No two individuals are alike, consequently a system of measurements have been useful in identifying criminals, but the adoption of the average system for this purpose would be folly. So it is in this instance.

Dr. Talbot in closing the discussion said that if the musles were the cause of the contracted arch, the first molar would be likely to be affected, which it never is.

Dr. L. E. Custer read a paper entitled, "The physiology of the action of obtundents."

The dentinal fiber is composed of simple protoplasm, with exalted sensibility. It contains albumen and is coagulable. It contains much water and does not entirely fill the dentinal tube, being surrounded by water. This water can be removed. The temperature can be reduced. There is no blood circulation, consequently anodynes introduced systemically cannot produce an effect rapidly. The successful agent for obtunding sensitive dentine, must not be a coagulant, must have an affinity for water, and a penetrative property exceeding the ordinary nutritive process. Change of structure is most easily and readily accomplished by coagulants. Extreme heat is capable of coagulating the fiber deeply. Penetrating escharotics, because their activity cannot be limited, are dangerous as they endanger the life of the pulp. Desiccation, by extracting the water from the tubuli of the dentine around the fiber and from the fiber itself as well, will destroy the function temporarily and consequently produce insensibility. The watery contents of the dentine is extracted by evaporation, and in this condition the application to the dentinal fiber of an agent having a strong affinity for water will extract its watery constituent. Many agents are used for this purpose, but absolute alcohol is the most acceptable and a representative. The water of the dentine is easily extracted, but the molecularly held water of the fiber is more difficult to abstract, hence the difficulty experienced in efforts to obtund sensitive dentine. Evaporation and affinity are the two great factors in dehydration. Complete insensibility is more easily obtained by dehydration than by coagulation, but the most profound effects are to be had by a combination of the two methods. In addition to dehydration, chloride of zinc, as a representative coagulant, is used to produce profound impressions. This method must necessarily be exceedingly painful. Change of temperature is another popular method of producing insensibility. Lowering of temperature will produce a corresponding lower neural activity until complete insensibility will have been obtained. Refrigeration is a more practical method of anæsthesia than the chemical methods. Volatile agents are used to produce refrigerating anæsthesia, such as sulphuric ether, chloride of methyl, rhigolene, nitrous oxide, etc. These agents do not dehydrate, but lower temperature. Owing to the fact that sufficient dehydration or coagulation cannot be had to obtain insensibility without endangering the life of the pulp, the most practical and safe method of obtunding sensitive dentine is by refrigeration.

The application of vaporizable agents in a warm or hot blast are essentially methods of desiccation. The heat being the only virtuous agent unless the medicament have an affinity for water. Alcohol in this connection is valuable because of its affinity for water, but the volatile oils have no such affinity and consequently little or no value.

There was no discussion of Dr. Custer's paper.

By suspension of the regular order of business, Dr. Corydon Palmer had hung upon the wall an excellent oil painting of Dr. W. H. Atkinson, which he uncovered and in a brief speech presented it to the association.

(To be continued.)

